# Draft genome sequences of four *Helicobacter pylori* isolates, from New Zealand

**DOI:** 10.1128/mra.00878-26

**Published:** 2026-10-13

**Authors:** Zoe King, Colleen M. Higgins, Kevin C. Lee, R. Brent Seale

**Affiliations:** 1 School of Science, Faculty of Health and Environmental Sciences, Auckland University of Technology 1410, Auckland, New Zealand; Fluxus Inc., Sunnyvale, California, USA

**Keywords:** *Helicobacter pylori*, antibiotic resistance, New Zealand

## Abstract

*Helicobacter pylori* is a bacterium linked with gastric ulcers and gastric carcinoma that has high instances of antibiotic resistance. We present here the draft genome sequences of four *H*. *pylori* isolates, from four patients who had undergone gastroscopy and biopsies that were processed at Middlemore Hospital Laboratory, Auckland, New Zealand.

## ANNOUNCEMENT

*Helicobacter pylori* is a Gram-negative bacterium associated with gastritis, gastric ulcers, and, in severe cases, gastric carcinoma (1–3). Due to the increasing rates of antibiotic resistance worldwide, it is becoming harder to effectively treat this infection (4, 5). Within New Zealand, antibiotic-resistant *H. pylori* are severely understudied, with little known on current resistant rates and underlying mechanisms of resistance.

This report announces the draft genome sequences of four *H*. *pylori* isolates (NZ_A, NZ_B, NZ_C, and NZ_E), isolated from four anonymized gastroscopy biopsies processed by Middlemore Hospital Laboratory, Auckland, New Zealand, in 2019. Biopsies were macerated and cultured on Anaerobic Agar plates (Fort Richard, NZ) for 10 days at 37°C under microaerophilic conditions (CampyGen, Oxoid). Isolates were preserved in Laboratoire de Santé Publique du Québec preservation medium (Fort Richard, NZ), revived on Mueller Hinton agar plates with 5% sheep blood (Fort Richard, NZ), and transported to Auckland University of Technology. Isolates were sub-cultured onto Columbia Sheep Blood agar (Fort Richard, NZ) at 37°C under microaerophilic conditions (Campy GasPak, Fort Richard, NZ) for 7–10 days before gDNA was extracted. Both the Health and Disability Ethics Committee (HDEC, NZ) and Auckland University of Technology Ethics Committee (AUTEC) determined that the project fell outside their ethical review scope.

Antibiotic susceptibility testing was performed at Middlemore Hospital Laboratory using E-test gradient diffusion minimum inhibitory concentration strips (bioMérieux, France) for amoxicillin, clarithromycin, tetracycline, and metronidazole following the European Committee on Antimicrobial Susceptibility Testing breakpoint guidelines (v7.1, 2017) (6). Isolate NZ_A was sensitive to all antibiotics; NZ_B and NZ_E were resistant to clarithromycin and metronidazole (>256 µg/mL); NZ_C was resistant to clarithromycin alone (24 μg/mL).

Genomic DNA was extracted using a DNeasy Blood and Tissue Kit (Qiagen, Germany) following the manufacturer’s instructions, followed by ethanol precipitation. Quality of gDNA (A260/A280 and A260/A230 ratios) and concentration were measured on a GE NanoVue spectrophotometer (GE Biosciences, USA) and Qubit 2.0 Fluorometer (ThermoFisher Scientific, USA), respectively. Libraries were prepared with a NEBNext Ultra DNA Library Prep Kit for Illumina (NEB, USA), as per the manufacturer’s instructions, then validated using an Agilent 2100 Bioanalyzer (Agilent Technologies, USA), and quantified with a Qubit 2.0 Fluorometer. Genomes were sequenced on an Illumina HiSeq 2500 (2 × 150 bp; Azenta Life Sciences, Suzhou, China). Reads were quality-filtered using bcl2fastq v2.17 (7), trimmed using AdapterRemoval v2.3.0 (8), and assembled using SPAdes v3.12.0 (9) via Unicycler v0.4.8 (10). Assembly coverage was calculated using BBMap v38.36 (11); N50 and additional metrics were extracted from the Unicycler assembly logs; and estimated completeness was determined by CheckM2 v1.1.0 (12) (Table 1). Default parameters were used for all software. Although still in draft form, assembly size, G + C content, and protein-coding gene counts are comparable to those of other complete *H. pylori* genomes (13, 14). Candidate plasmid contigs in isolates NZ_A, NZ_C, and NZ_E (pHPA001, pHPC001, and pHPE001) were identified from Bandage v0.8.1 (15) visualization as single circular contigs of 2,993–4,212 bp, within typical *H. pylori* plasmid sizes. NCBI BLASTn search (https://www.ncbi.nlm.nih.gov/) of the plasmid contigs showed high similarity to other *H. pylori* plasmids. Assemblies were annotated using the NCBI PGAP (16) with default parameters.

**TABLE 1:** Draft genome features of *H. pylori* isolates NZ_A, NZ_B, NZ_C, and NZ_E

Genome feature	Isolates			
	NZ_A	NZ_B	NZ_C	NZ_E
Number of read pairs	5,672,487	5,104,579	4,905,426	5,599,851
Genome size (bp)	1,624,747	1,669,002	1,553,684	1,632,396
Number of contigs	33	38	35	48
G + C content (%)	39	38	39	39
N50	136,419	215,027	145,451	65,734
Genome coverage (×)	1,039	906	942	1,020
Protein coding genes	1,454	1,489	1,406	1,457
rRNA genes (5S, 16S, 23S rRNA)	3 (1, 1, 1)	3 (1, 1, 1)	3 (1, 1, 1)	2 (0, 1, 1)
CheckM2 completeness (%)	100	100	100	99.99
CheckM2 contamination (%)	0.05	0.01	0	0.19
Genome accession number	JAFCJA000000000	JAFCJB000000000	JAFCJC000000000	JAFCJD000000000
BioSample accession number	SAMN17734761	SAMN17734762	SAMN17734763	SAMN17734764
SRA accession number	SRR39611657	SRR39611656	SRR39611655	SRR39611654

## Data Availability

The genomes were uploaded to the National Centre for Biotechnology Information (NCBI) database under BioProject number PRJNA698714 and assigned unique accession numbers as provided in Table 1.
